# A simple clinical score to identify likely hepatitis B vaccination non-responders – data from a retrospective single center study

**DOI:** 10.1186/s12879-020-05634-y

**Published:** 2020-11-25

**Authors:** Marc A. Meier, Christoph T. Berger

**Affiliations:** 1 Vaccination Clinic, Medical Outpatient Unit, University Hospital Basel, Petersgraben 4, 4031 Basel, Switzerland; 2 Translational Immunology, Department of Biomedicine, University of Basel, Basel, Switzerland

**Keywords:** Hepatitis B, Vaccine, Non-response, HBs, Antibodies, Smoking, Age, BMI, Risk score

## Abstract

**Background:**

About 10% of Hepatitis B vaccinated individuals mount no protective antibody levels against the hepatitis B surface antigen (HBs-Ag). Older age at primary immunization, obesity and smoking have previously been reported as risk factors associated with vaccine non-response. Here we tested whether these factors alone may allow selecting subjects that benefit from individualized immunization schedules.

**Methods:**

Retrospective database analysis screening > 15,000 individual anti-HBs-IgG measurements. Non-responders (NR; anti-HBs-IgG < 10 IU/L) and low-responders (LR; anti-HBs-IgG 10–100 IU/L) were identified. Vaccine type, demographics, lifestyle, and immunological factors (leucocyte subset counts) were compared between NR, LR, and responders (R).

**Results:**

We identified 113 LR/NR and compared them to 134 vaccine responders. We confirmed higher median age at primary vaccination (24.0 (R) vs. 30.5 (NR) vs. 31 (LR) years, *p* = 0.001), higher median BMI (23.2 kg/m^2^ (R) vs. 23.4 kg/m^2^ (NR) vs. 25.1 kg/m^2^ (LR), p = 0.001) and being a smoker (% smokers: 30.8% (R) vs. 57.1% (NR) vs. 52.5% (LR), *p* = 0.01) as factors negatively associated with anti-HBs-IgG levels. In a ROC analysis including these factors in a 6-point score, a high score predicted non-response with a specificity of 85% but at low sensitivity (47%).

**Conclusion:**

A simple clinical risk score based on age, obesity, and smoking identifies individuals with a high likelihood of vaccine failure. Non-responders with a low score are candidates for in-depth analyses to better understand the immunological causes of HBV vaccine non-response.

**Supplementary Information:**

The online version contains supplementary material available at 10.1186/s12879-020-05634-y.

## Background

Chronic Hepatitis B virus (HBV) infection affects about 400 million individuals worldwide, and cumulatively accounts for about 1 million deaths annually [1]. Vaccination against HBV is safe and very effective in preventing HBV infection and its complications [2]. Since the early 1990s, high-risk populations -especially health care workers- have been systematically vaccinated. More recently, vaccination efforts have been extended and many national vaccination programs now recommend HBV vaccination for the general population. The currently available HBV vaccine preparations use recombinant hepatitis B surface antigen (HBsAg) as the antigen. Vaccination induces an antibody-based protective immunity targeting the HBsAg [3, 4].

Primary immunization protects at least two decades in the vast majority of immunocompetent individuals [5]. An anti-HBs antibody titer above 100 IU/L is considered protective, whereas titers below 10 IU/L confer no reliable protection from infection. Accordingly, vaccine recipients are graded into ‘non-responders’ (< 10 IU/L), ‘low-responders’ (10–100 IU/L), and ‘responders’ (> 100 IU/L), respectively [6]. Large vaccination studies suggested non-responder rates in the range of 5 to 10% of all HBV vaccinated healthy individuals vaccinated using the standard vaccination regimen [2]. Several epidemiological studies have established that age at immunization, overweight, gender, smoking status, co-morbidities and immunosuppression affect the vaccine response [6–15]. In many of the subjects with these ‘risk factors’, protective immunity can be achieved by repeated additional doses (‘booster’) [3]. The reason for the failure to adequately respond to HBV vaccination in subjects without classical risk factors has been attributed to genetic factors (MHC), reduced T cell activation or failure of the T cells to recognize the HBs Ag (‘hole in the repertoire’) [16]. Here, we studied a group of hepatitis B vaccine non-responders and tested, if a simple score based on age, and -the potentially modifiable life-style factors- BMI and smoking status may allow selecting subjects with a high likelihood for vaccine failure.

## Methods

### Vaccine recipient cohorts and screening strategy

The local Ethical committee (EKNZ-265/12) approved the study. We performed a retrospective database screening of HBs-IgG test results collected between 1995 and 2012. We limited our analysis on ‘potential non-responders’ from the medical outpatient clinic, the vaccination clinic and occupational health service (OHS), in order to exclude subjects with relevant comorbidities. We excluded subjects with only a single anti-HBs-IgG measurement, and we divided the remaining into ‘potential non-responders’ (all measurements < 100 IU/L) vs. ‘responders’ (at least one measurement > 100 IU/L). For the patient’s chart review of all ‘potential non-responders’ we excluded subjects with (i) no chart available; (ii) incomplete primary vaccination schedules; (iii) obvious immunosuppression (i.e. chemotherapy, HIV, active cancer, immunosuppressive drugs); (iv) documented titers > 100 IU/L in the chart, (v) age > 60 at primary immunization, or (v) natural HBV infection. As a control group, 134 subjects with well-documented vaccination schedules and demographic factors were selected from the responder pool. The screening and subject selection process is summarized in Figure S1.

Data collection for all studied subjects included: anti-HBs-IgG levels, vaccine preparation/brand, vaccination schedule, demographic and lifestyle factors (age, gender, BMI, smoking habits). Besides, total white blood cell count (WBC), neutrophil count, and lymphocyte count were also extracted from the charts, if these were available within a time window of 6 months before and/or after vaccination. Mean values were calculated in cases where several time points were available within the defined time window.

### Statistical analyses

We compared continuous variables between two groups using an unpaired t-test. Binary data were analyzed using chi-square tests. For comparisons of more than two groups one-way ANOVA (Kruskal-Wallis) was used followed by correction for multiple comparisons (Dunn’s correction). Logistic regression analysis was performed using JMP software (V11, Institute). All statistical analyses, with the exception of the logistic regression, were performed using Prism Software (V6.0b, GraphPad Software).

## Results

### Characterization of vaccine responders and non-responders

We identified 40 ‘non-responders’ and 73 ‘low responders’ that we compared to 134 ‘responders’. Anti-HBs-IgG levels in the responders were highly variable covering, almost 2.5 log differences between the extremes (Figure S2). Non-responders received a median of six HBV vaccine doses (range 3–12) compared to four (range 3–7) in the responders (*p* < 0.001). Notably, 23.7% of the non-responders and 23.3% of the low-responders received seven or more vaccinations (Figure S2).

**Fig. 1:**
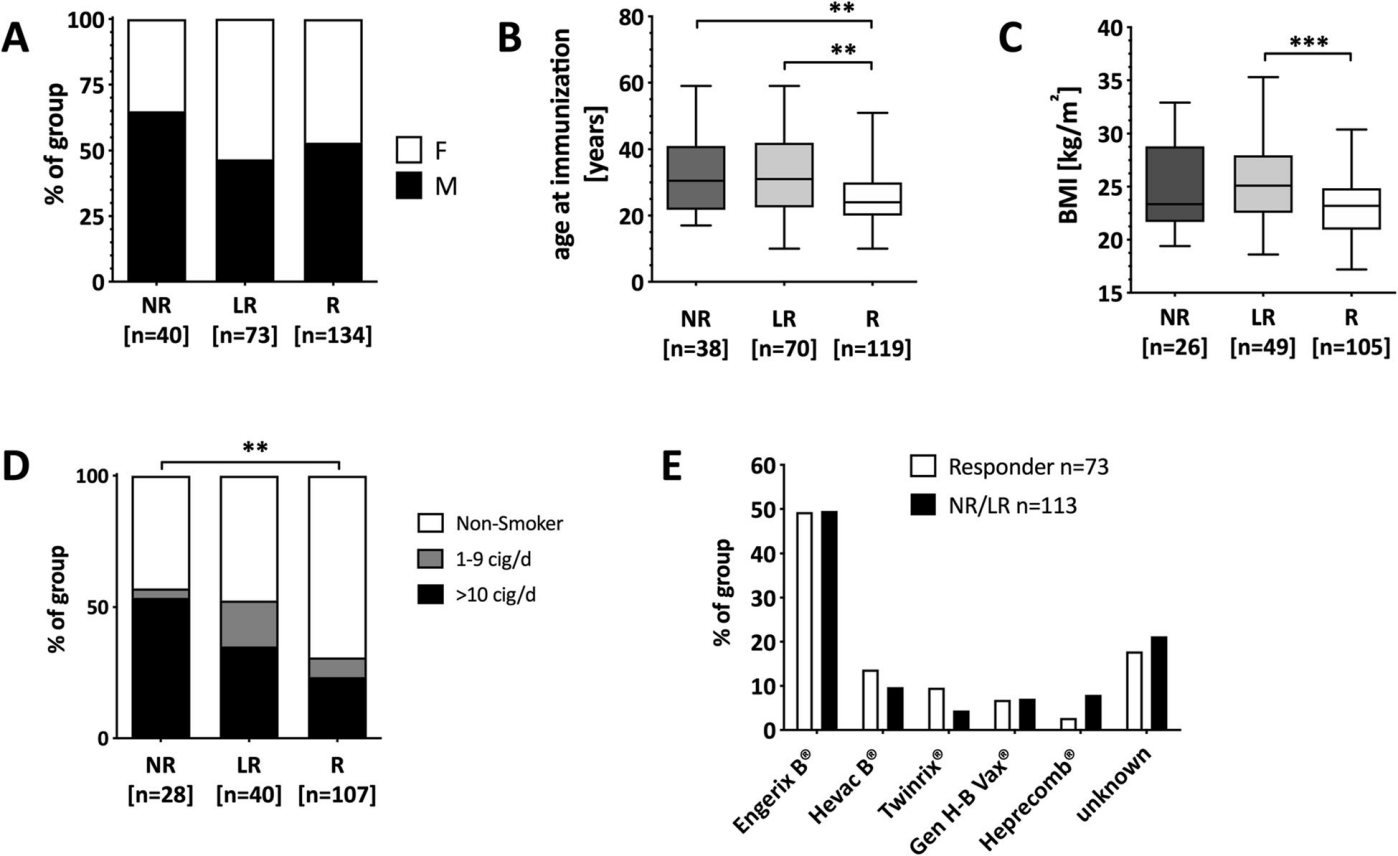
Association of demographic and lifestyle factors with vaccine response status. Distribution of sex in % within each group (**a**), age at the first HBV vaccination (**b**), Body Mass Index (BMI) (**c**), and % smokers per group (**d**) is shown. Kruskal-Wallis test was used to compare the three groups. Smokers were subdivided into light (0–9 cigarettes/day; grey) or heavy (≥10 cigarettes/day; black) smokers. (**e**) Distribution of vaccine preparation/type in responders (white bars) and low−/non-responders (black bars). **p < 0.01, ***p < 0.001

Gender distribution was equal between the three groups (Fig. 1a and Table S1). At the time of the basic immunization, non- and low-responders were significantly older than the responders (Fig. 1b). BMI was significantly higher in low-responders compared to responders (median 25.1 kg/m^2^ vs. 23.2 kg/m^2^, *p* < 0.001), but not between the responder and the non-responder group (Fig. 1c). About 1 out of 3 non-responders (29.6%) and 1 out of 4 low-responders (24.5%) had a BMI above 28 kg/m^2^, while this was only the case in 4.7% of the responders. Low- and non-responders were comparably enriched for smokers, but smokers in the non-responder group were almost exclusively ‘heavy smokers’, defined as ≥10 cigarettes per day (Fig. 1d).

Various vaccine types were available during the study period (Table S1). Data on the vaccine used for the baseline immunization was available for all non-responders (*n* = 113) and 59% of the responders (*n* = 73). We found that the vaccines used in the groups were very comparable, with Engerix® being the most frequently applied in both groups (Fig. 1e). The only vaccine that was used more frequently in the non-responder group (8% vs. 2.7%) was Heprecomb®. Notably, Heprecomb® contains a low HBs antigen dose and is the preparation with the lowest adjuvant content (aluminum hydroxide) (Table S2).

Leucocyte or lymphocyte counts were only available for a small subset of the cohort (*n* = 65 for the leucocyte counts; *n* = 38 for the lymphocyte counts), preventing meaningful analyses. We found, however, that neither relative leucopenia, neutropenia, or lymphopenia were associated with vaccine non-response. Contrarily, the low−/non-responders had the highest leucocyte, neutrophil, and lymphocyte counts (Fig. S3*)*.

In summary, the analysis of the demographic and lifestyle factors on the vaccine response confirmed a negative impact of age at immunization, overweight, and smoking.

### High specificity, but low sensitivity of a risk score to predict non-response

**Fig. 2:**
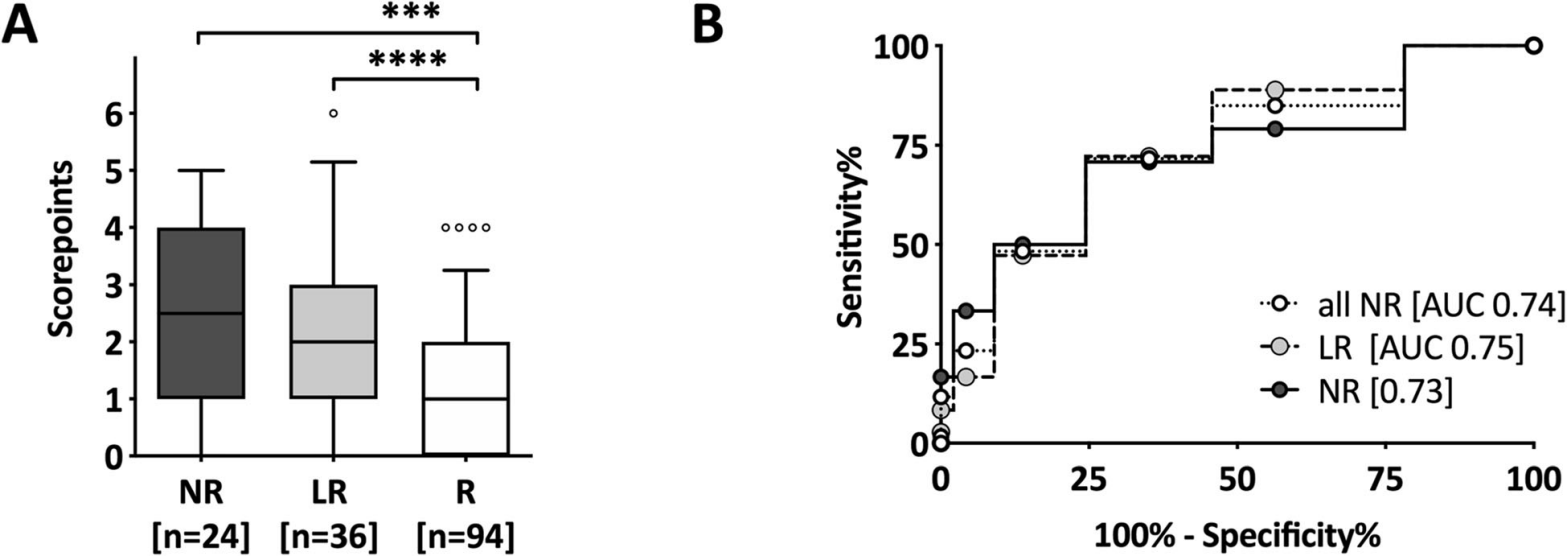
Specificity and sensitivity of a risk factor-based score for HBV vaccine responder status. An individual risk score (0–6 points) was calculated for each subject: i) BMI: normal BMI (0 points), BMI > 25–30 kg/m^2^ (1 point), and BMI > 30 kg/m^2^ (2 points); ii) Age: < 30 (0 points), 30–40 (1point), and > 40 (2 points); and iii) smoking status: non-smoker (0 points), 1–9 cigarettes a day (1 point), and > 9 cigarettes a day (2 points). Only subjects with complete datasets regarding these risk factors (*n* = 154: 24 NR (black), 36 LR (grey), 94 R (white)) were considered for this analysis. **a** Average score points in three groups are displayed. Box indicates median and IQR; whiskers indicate 5-95th percentile. **b** Receiver operating characteristic (ROC)- curve and AUC (Area under curve) of risk scores are displayed. Non-responders are shown in black, low-responders in grey and responders in white

We next tested if a risk score would identify subjects with a high risk of HBV vaccine non-response. Logistic regression analysis confirmed the negative impact of the three risk factors: age, smoking and high BMI. BMI showed the strongest association (*p* = 0.0005), followed by smoking status (*p* = 0.011), and age at vaccination (*p* = 0.022). We calculated a score using a study sample subset for which data on all of these three parameters were available (i.e. R = 94, NR = 24, LR = 36). Each variable was categorized in three clinically relevant groups: i) normal BMI (0 points), BMI > 25–30 kg/m^2^ (1 point), and BMI > 30 kg/m^2^ (2 points); ii) Age at primary hepatitis B immunization < 30 (0 points), 30–40 (1point), and > 40 (2 points); and iii) non-smoker (0 points), 1–9 cigarettes a day (1 point), and ≥ 10 cigarettes a day (2 points), resulting in a potential score between 0 and 6. Vaccine responders had a median score of 1 (IQR 0–2), low-responders a median score of 2 (IQR 1–3), and non-responders a median score of 2.5 (IQR 1–4) (Fig. 2a). Only about 5% of the responders, but 35% of the low−/non-responders had a score of 4 or higher. In contrast, about 85% of the responders had a score of 2 or less, while this was the case in less than half of the low−/non-responders combined. In a ROC curve, a cut-off of ≥2 score points yielded a sensitivity of 71.7% and specificity of 64.9%. Specificity was high (86%) at a higher cut-off of ≥4 score points at the cost of a low sensitivity (47%) (Fig. 2b).

When we performed the same analysis separately for subjects that received ≤4 vs. those who received ≥5 vaccinations, we found that the AUC was substantially lower in those who received ≥5 vaccinations (0.79 vs. 0.66) (data not shown). This suggests that in subjects that mount no protective titers following repeated boostering (i.e. ≥5 vaccinations) the risk factors age, BMI, and smoking status were less relevant.

## Discussion

HBV vaccination results in protective antibody titers in about 90% of the vaccinated subjects. Anti-HBs antibody titer measurements are clinically available, but due to low cost-effectiveness only recommended for high-risk populations, specifically health care workers. However, on an individual basis, every vaccinated subject would want to know whether they are fully protected. Recent epidemiological data indicate that currently, other populations than health care workers are at the highest risk to contract HBV [17]. Hence, there is a need to identify non-responders that could be specifically tested and vaccinated using individualized vaccination protocols [7]. Overall, this would allow increasing the population coverage of immunity and thereby reducing the disease prevalence in a population.

Here we performed a retrospective, single-center study to test whether age, BMI and smoker status could be used -alone or in combination- to predict HBV vaccination non-response. We confirmed that higher age at vaccination, smoking, and a high BMI associated with a higher risk for non-response [10–14]. About 1 out of 3 non-responders (or 29.6%) and 1 out of 4 low-responders (24.5%) had a BMI above 28 kg/m^2^, while this was only the case in 4.7% of the responders. There are several potential explanations for this finding: i) the amount of HBs Ag is too low in relation to the body mass, ii) there is a substantial immunological dysfunction in the obese [18], or iii) the needles are too short to reach the muscle, thus resulting in subcutaneous vaccination associated with blunted responses [7]. The negative effect of older age, may be explained with the progressive immune dysfunction occurring in an aging organism. Interestingly, this immune dysfunction seems to be evolving already in middle-aged subjects, as we excluded all vaccinees older than 60 years. Finally, we confirmed that smoking seems to have a strong and dose-dependent impact on HBV vaccine response in a larger series of non−/low-responders [11, 14]. In contrast to others, we observed an equal gender distribution in responders and non-responders. Concomitant disease has been suggested as another risk factor for lower seroconversion to the HBV vaccine. In our study, we focused on subjects < 60 years old without obvious immunosuppression. Since co-morbidities were not prospectively assessed, we cannot exclude they may have contributed to vaccine failure in some instances.

Using quantification of leucocytes and leucocyte subsets, we could exclude that absolute (immune cell deficiency) or relative immunocytopenia (low numbers within the normal range for any of the immune cell subsets) are frequent causes of poor vaccine response status. It is, however, of little surprise that such crude measurements fail to reflect non-response. Thus, more in-depth immunological analyses would be necessary that should preferentially be performed on non-responders lacking any of the other risk factors for non-response.

We calculated a very simple risk score integrating the three identified risk factors: age, BMI and smoker status. A high score (≥4 points) allowed the identification of non-responders with high specificity (86.2%), but at the cost of low sensitivity (46.7%). In other words, if the score would be calculated at the time of vaccination and HBs-IgG would be determined after primary immunization, we would expect that 17 out of 20 tested subjects with a score ≥ 4 would be LR or NR. Thus, our risk score could be used to detect at least a subgroup of non-responders. This finding may justify targeted anti-HBs-IgG measurements and individualized vaccination strategies in these selected subjects with a high score. That the risk score imperfectly predicts non-responders is not surprising, as additional risk factors such as HLA type or hyporeactive T cells have been described as risk factors for HBV vaccination non-response, but were not addressed in our study.

Different strategies have been suggested to enhance the immune response to hepatitis B vaccination, including additional booster doses, an increased HBs antigen dose, intradermal vaccinations or the use of adjuvanted vaccines [7, 19–21]. Three doses of the 3′-deacylated monophosphoryl lipid A (3D-MPL) and alum containing AS04-adjuvanted HBV vaccine resulted in seroprotection in 98% of previous non-responders (HBs-IgG < 10 U/L) compared to only 68% in subjects that received three additional standard vaccine doses [21]. Very recently, a Dutch multicenter study compared the efficacy of additional three doses (0, 1, 2 months) of either the standard HBV vaccine, an HBV/HAV combination vaccine, a double dose vaccine (40 μg HBsAg) or the AS04-adjuvanted vaccine (20 μg HBsAg) in previous non-responders. Percentage of responders was 67% in the standard vaccine group compared to 80% in the HBV/HAV vaccine group, 83% double dose group and 87% in the AS04-adjuvanted group [22]. Whether subjects at risk for non-response identified by our score should be immunized using one of these alternative strategies remains to be tested prospectively.

The main limitations of the study include the retrospective design resulting in incomplete data collection, heterogenous time points for the anti-HBs-IgG measurements following vaccinations, and the rather small, not perfectly matched control population. A validation of the score in a larger cohort or a prospective study is warranted, since we only applied it to the training cohort. Moreover, the study population that included subjects seen at a University Hospital as well as health care workers may not be representative of other populations.

A major strength of the study is that, by using a retrospective study design, we were able to identify a substantial number (*n* = 113) of HBV vaccine non−/low-responders screening more than 15′000 HBV titer measurements. In most previous studies on HBV non-response focus was on antibodies after primary vaccination. In our cohort non-responders received in average six vaccinations, but some up to 14. Hence, this population represents true non-responders and may potentially be enriched for subjects with immunological or genetic factors (rather than BMI, age and smoking) driving non-response.

## Conclusions

Taken together, our data provide a rational to test post-vaccination anti-HBs-IgG in older, obese smokers and efforts should be put into increasing response rates in these subjects [7, 23]. At the same time, the low sensitivity of a high cut-off (i.e. ≥4) strongly points at other risk factors for non-response that are likely of immunological nature. Future immunological studies on HBV vaccine non-response should focus on young, non-obese non-smokers since most likely immunological factors are the cause for the failure to respond despite repeated vaccinations in this population.

## Supplementary Information

**Additional file 1:** **Table S1.** Demographics and anti-HBs levels. **Table S2.** Characteristics of the vaccine types used in the study population. **Figure S1**. Algorithm for the identification of HBV vaccine non-responders. **Figure S2.** Vaccine specific antibody titer distribution and number of vaccinations received per group. **Figure S3**. Reduced vaccine responses were not linked to low immune cell counts in vaccine recipients.

## Data Availability

All data will be made available by the corresponding author upon request.

## Abbreviations

HBV: Hepatitis B Virus
HBs Ag: Hepatitis B surface antigen
NR: (Hepatitis B vaccine) non-responders
LR: (Hepatitis B vaccine) low-responders
R: (Hepatitis B vaccine) responders
BMI: Body Mass Index
WBC: White Blood Count
HLA: Human Leucocyte Antigen
MHC: Major Histocompatibility Complex
ANOVA: Analysis of variance
ROC curve: Receiver operating characteristic curve
AUC: Area under the curve
HAV: Hepatitis A Virus
