# Supplementary material for: A simple clinical score to identify likely hepatitis B vaccination non-responders – data from a retrospective single center study
Source: BMC Infect Dis. 2020 Nov 25;20:891. doi: 10.1186/s12879-020-05634-y (PMC7690196; doi:10.1186/s12879-020-05634-y)
Supplement: Supplementary file 1 — Additional file 1: Table S1. Demographics and anti-HBs levels. Table S2. Characteristics of the vaccine types used in the study population. Figure S1. Algorithm for the identification of HBV vaccine non-responders. Figure S2. Vaccine specific antibody titer distribution and number of vaccinations received per group. Figure S3. Reduced vaccine responses were not linked to low immune cell counts in vaccine recipients. [file 12879_2020_5634_MOESM1_ESM.docx]

**Supplementary Data – Meier et al. ‘A simple clinical score to identify likely hepatitis B vaccination non-responders – Data from a retrospective single center study’**

**Supplementary Table S1: Demographics and anti-HBs levels**

|  | **Responders** | **Low-responders** | **Non-responders** |
| --- | --- | --- | --- |
| Subjects, n | 134 | 71 | 40 |
| Age median, median (IQR) | 24 (20-30) | 31 (22.5-42) | 30.5 (21.8-41) |
| Female, No. (%) | 47% | 53% | 35% |
| BMI, median (IQR) | 23.2 (21-24.9) | 25.1 (22.6-28) | 23.4 (21.7-28.8) |
| Non-smokers (%) | 69.2 | 47.5 | 42.9 |
| < 10 cigarettes per day | 7.5 | 175 | 3.6 |
| ≥ 10 cigarettes per day | 23.4 | 35 | 53.6 |
| n HBV doses received | 4 (3-4) | 5 (4-6) | 6 (4.7-6.3) |
| 1st anti-HBs IgG IU/L | n/a | 17 (5-49) | 1.4 (0-3.8) |
| peak anti-HBs IgG IU/L | 3896 (1000-15000) | 48 (23-78) | 2 (1-8) |
|  |  |  |  |

**IQR= interquartile range; n= number**

**Supplementary Table S2: Characteristics of the vaccine types used in the study population**

|  | Engerix^©^ | Gen-H-B-Vax^©^ | Heprecomb^©^ | Hevac B^©^ | Twinrix^©^ |
| --- | --- | --- | --- | --- | --- |
| Manufacturer | GSK | Sanofi  Pasteur MSD | Berna Biotech | Sanofi  Pasteur | GSK |
| Vaccine schedule (months) | 0 – 1 – 6 | 0 – 1 – 6 | 0 – 1 – 6 | 0 – 1 – 2 | 0 – 1 – 6 |
| HBs Ag content | 20 μg | 10 μg | 10 μg | 5 μg | 20 μg |
| Adjuvants | 0.95 mg  Al(OH)_3_ | 0.5 mg  AlPO_4_ | 0.25 mg  Al(OH)_3_ | 1.25 mg  Al(OH)_3_ | 0.45 mg  Al(OH)_3_ |
| HBs Ag origin | Recombinant  (Yeast) | Recombinant  (Yeast) | Recombinant  (Yeast) | Plasma-derived | Recombinant  (Yeast) |

**Supplementary Figure Legends**

**
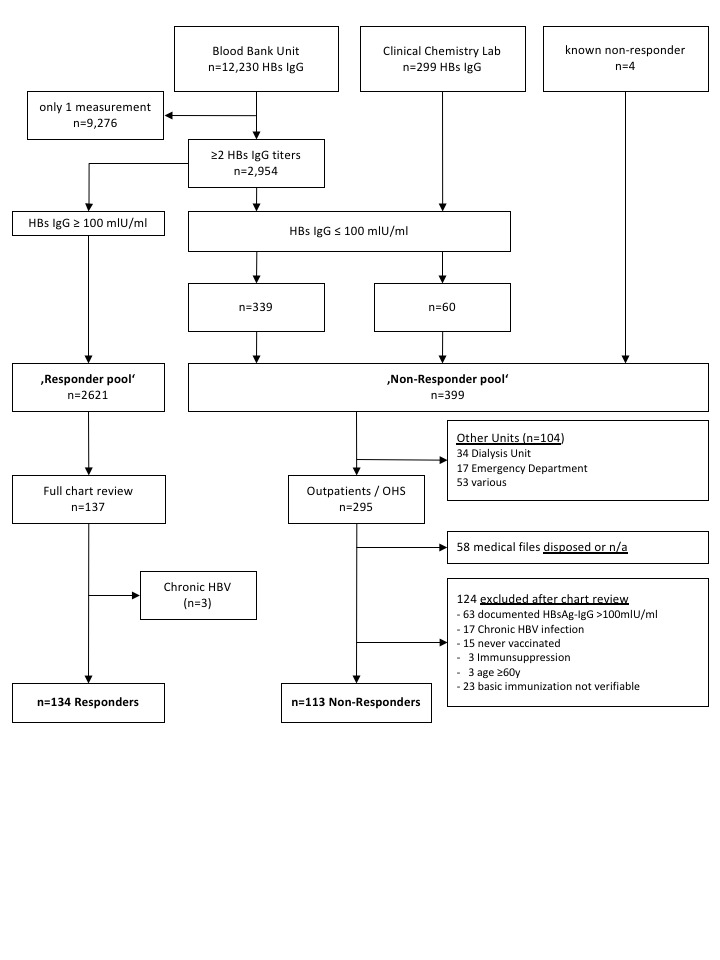
**

**Figure S1: Algorithm for the identification of HBV vaccine non-responders.** Reasons for exclusion of subjects are indicated. OHS= occupational health service; n/a= not available.

**
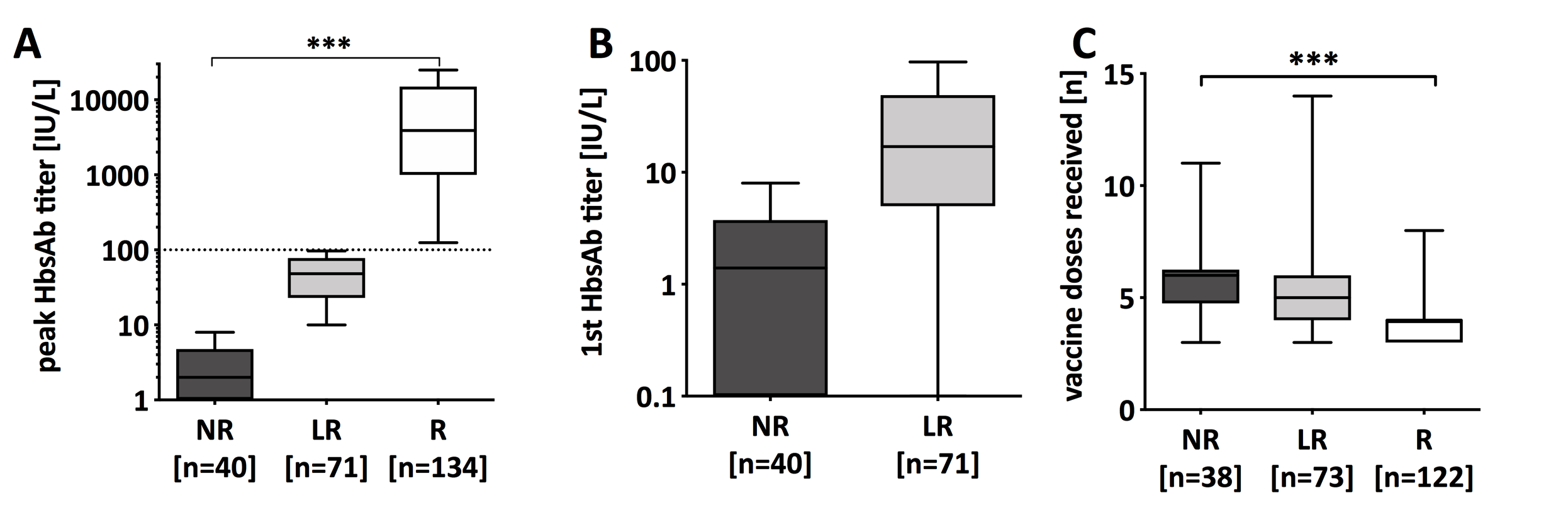
**

**Figure S2: Vaccine specific antibody titer distribution and number of vaccinations received per group.** (A) Distribution of the highest measured anti-HBs-IgG in non-responders (NR), low-responders (LR) and responders (R). (B) The first available HBs IgG titer in the non-responder and in the low-responder group is indicated. Data indirectly indicates that some non-responders were able to mount responses between 10 and 100 IU/L following additional booster vaccinations. (C) Number of vaccines received in the three groups are compared. ***p<0.001

**
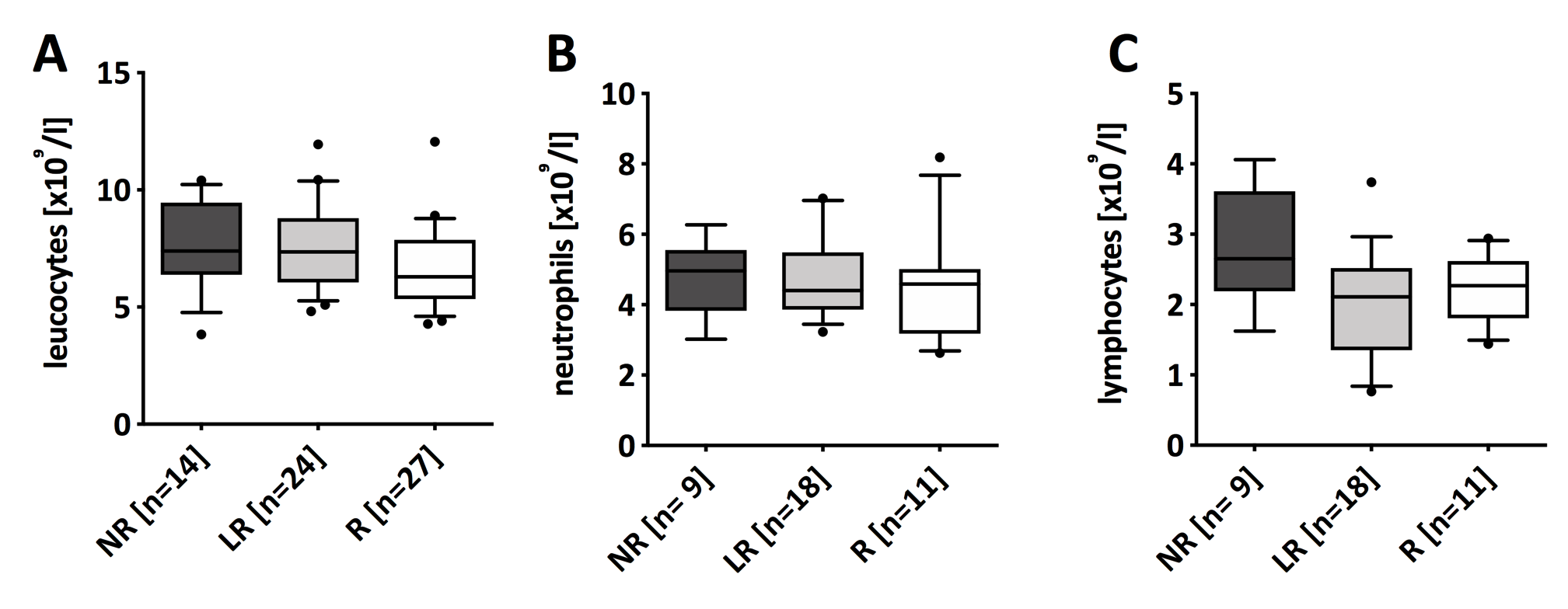
**

**Figure S3: Reduced vaccine responses were not linked to low immune cell counts in vaccine recipients.** For a subset of the study subjects (14/40 of NR (35%), 24/71 LR (34%) and 27/134 R (20%)) blood counts were available within 6 months of vaccination. Total leucocyte counts (A), neutrophil counts (B), and lymphocyte counts (C) were comparable between the three groups. Kruskal-Wallis test.
